# Cesarean delivery and associated socioeconomic factors and neonatal survival outcome in Kenya and Tanzania: analysis of national survey data

**DOI:** 10.1080/16549716.2020.1748403

**Published:** 2020-04-29

**Authors:** Malachi Ochieng Arunda, Anette Agardh, Benedict Oppong Asamoah

**Affiliations:** Social Medicine and Global Health, Department of Clinical Sciences, Lund University, Malmö, Sweden

**Keywords:** Socioeconomic factors, low-resource countries, cesarean delivery, logistic regression, neonatal mortality

## Abstract

**Background**: The increasing trends in cesarean delivery are globally acknowledged. However, in many low-resource countries, socioeconomic disparities have created a pattern of underuse and overuse among lower and higher socioeconomic groups. The impact of rising cesarean delivery rates on neonatal survival is also unclear.

**Objective**: To examine cesarean delivery and its associated socioeconomic patterns and neonatal survival outcome in Kenya and Tanzania.

**Methods**: We employed binary logistic regression to analyze cross-sectional demographic and health survey data on neonates born in health facilities in Kenya (2014) and Tanzania (2016).

**Results**: Cesarean delivery rates ranged from 5% among uneducated, rural Tanzanian women to 26% among educated urban women in Kenya to 37.5% among managers in urban Tanzania. Overall findings indicated higher odds of cesarean delivery among mothers from richest households, adjusted odds ratio (aOR) 1.4 (95% CI 1.2–1.8), those insured, aOR 1.6 (95% CI 1.3–1.9), highly educated, aOR 1.6 (95% CI 1.2–2.0) and managers aOR 1.7 (95% CI 1.3–2.2), compared to middle class, no insurance, primary education and unemployed, respectively. Overall, compared to normal births and while adjusting for maternal risk factors, cesarean delivery was significantly associated with neonatal mortality in Kenya and Tanzania, overall aOR 1.7 (95% CI 1.2–2.7). However, statistical significance ceased when fetal risk factors and number of antenatal care visits were further controlled for, aOR 1.6 (95% CI 0.9–2.6).

**Conclusion**: Disproportionate access to cesarean delivery has widened in Kenya and Tanzania. Higher risks of cesarean-related neonatal deaths exist. Medically indicated or not, the safety and/or choice of cesarean delivery is best addressed on individual basis at the health-facility level. However, policy initiatives to eliminate incentives, improve equitable access and accountability to reduce unnecessary cesarean deliveries through well-informed decisions are needed. Efforts to prevent unintended pregnancies among adolescents as well as training of health workers and continuous research to improve neonatal outcomes are vital.

## Background

The increasing trends of cesarean delivery (CD) are globally acknowledged [1–3]. However, socioeconomic inequities in many low- and middle-income countries (LMIC) appear to have created a pattern of underuse and overuse based on income and levels of education [2,4]. The impact of cesarean delivery trends on neonatal survival has also not been adequately examined [5–7]. A recent multi-country study estimated a tripling of CD rates since 1990 to 19% in 2014 with wide variations among and within regions and countries [1]. Estimated rates in Latin America and the Caribbean varied from 5% to 58% while rates in high-income countries (HIC) in the Nordics ranged between 15% and 27% [1,2,8]. Whereas the World Health Organization (WHO) emphasizes access to CD for all mothers in medical need, the organization’s 2015 review found that an optimal population-level CD rate should not exceed 10–15% based on medical indication [9]. Studies by Betrán et al. and Boatin et al. recommended increased access to CD in sub-Saharan Africa due to low CD rates, high maternal death rates, and slowly declining rates of newborn deaths within the first month, i.e. neonatal mortality rates (NMR) [1,2]. However, recent UNICEF country reports from certain sub-Saharan (SSA) countries including Kenya and Tanzania reveal unusual trends. The reports indicate comparatively higher rates of CD and disappointingly low declines in neonatal mortality rates among higher socioeconomic (SE) groups, despite higher coverages of both pre- and postnatal care and skilled birth assistance among these subpopulations [10–12]. New WHO recommendations such as 8+ antenatal visits [13] will expedite reduction of NMR to achieve target 2 of the Sustainable Development Goal 3 [14]. However, monitoring the impact of country-specific trends of CD rates and subsequent policy adjustments might sustain neonatal survival gains.

Cesarean delivery (or C-section) is an obstetric surgical procedure meant to save the life of a mother and her baby. Breech presentation, antepartum hemorrhage, fetal distress, prolonged and obstructed labor, placenta previa and other life-threatening medical indications require CD for safe delivery [5–7]. However, most of the rising elective CD rates among low-risk births in many LMIC are due to maternal request or physicians’ preference without plausible clinical indications [15–17]. In HIC such as Sweden, childbirth fear has also been associated with CD [18]. Elective CD has been associated with sepsis and respiratory problems, which are major causes of neonatal deaths globally [19]. While cesarean delivery has prevented many adverse pregnancy outcomes, the quality and conditions under which some procedures (both elective and emergency) are executed in many low-resourced settings have also resulted in many morbidities [20,21] and preventable mortalities [5,22–28]. The trade-offs between morbidities and benefits are generally unclear but also costly for weak health-care systems [29,30]. A cohort study in South America reported a significant increased risk of neonatal death among elective cesarean deliveries [28]. Another study in the USA also indicated a two-fold rise in neonatal deaths among CD-newborns without medical indication even after adjusting for key confounders [31]. Similarly, recent enquiry into maternal deaths in South African health facilities revealed 3 times higher risk of maternal deaths among CD births [24]. A systematic review in LMIC also found similar adverse neonatal outcomes after CD [24].

In many low-resourced settings, inadequate record-keeping makes it difficult to determine whether the adverse pregnancy outcomes occurred before birth or intrapartum or because of the CD procedure itself [29,32]. A study in five low-income countries (LIC) in SSA and Southeast Asia (SEA) found that 40% of health facility records had no CD fetal outcome information [6]. Nonetheless, although inadequate access to CD and delays by the expectant mothers to seek or reach care clearly have adverse impacts [33,34], incomplete records have also concealed emergency challenges of health facilities and impeded improvements in care as well as accountability [6,35–37]. Higher neonatal deaths associated with CD are reported in SSA than any other region [21]. It should be noted, however, that audits of a few upgraded and well-funded health facilities in SSA including Tanzania have reported reduction of both unnecessary CD and CD-related neonatal deaths [32,38].

**Figure 1.**
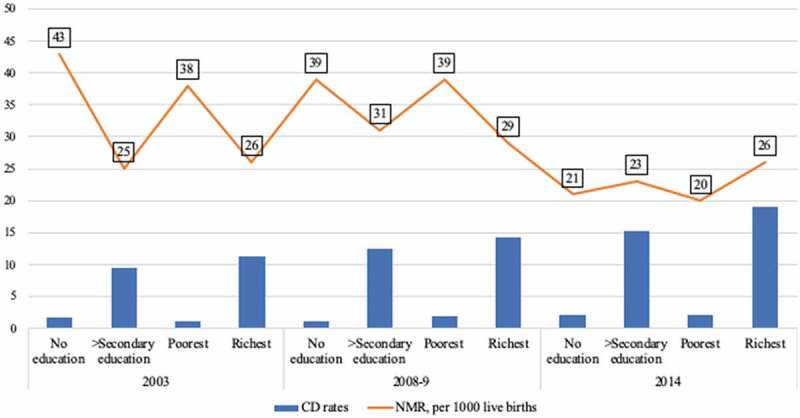
Neonatal mortality rates (NMR) and cesarean delivery (CD) rates among highest and lowest socioeconomic groups in Kenya between 2003 and 2014 [11,39]

**Figure 2.**
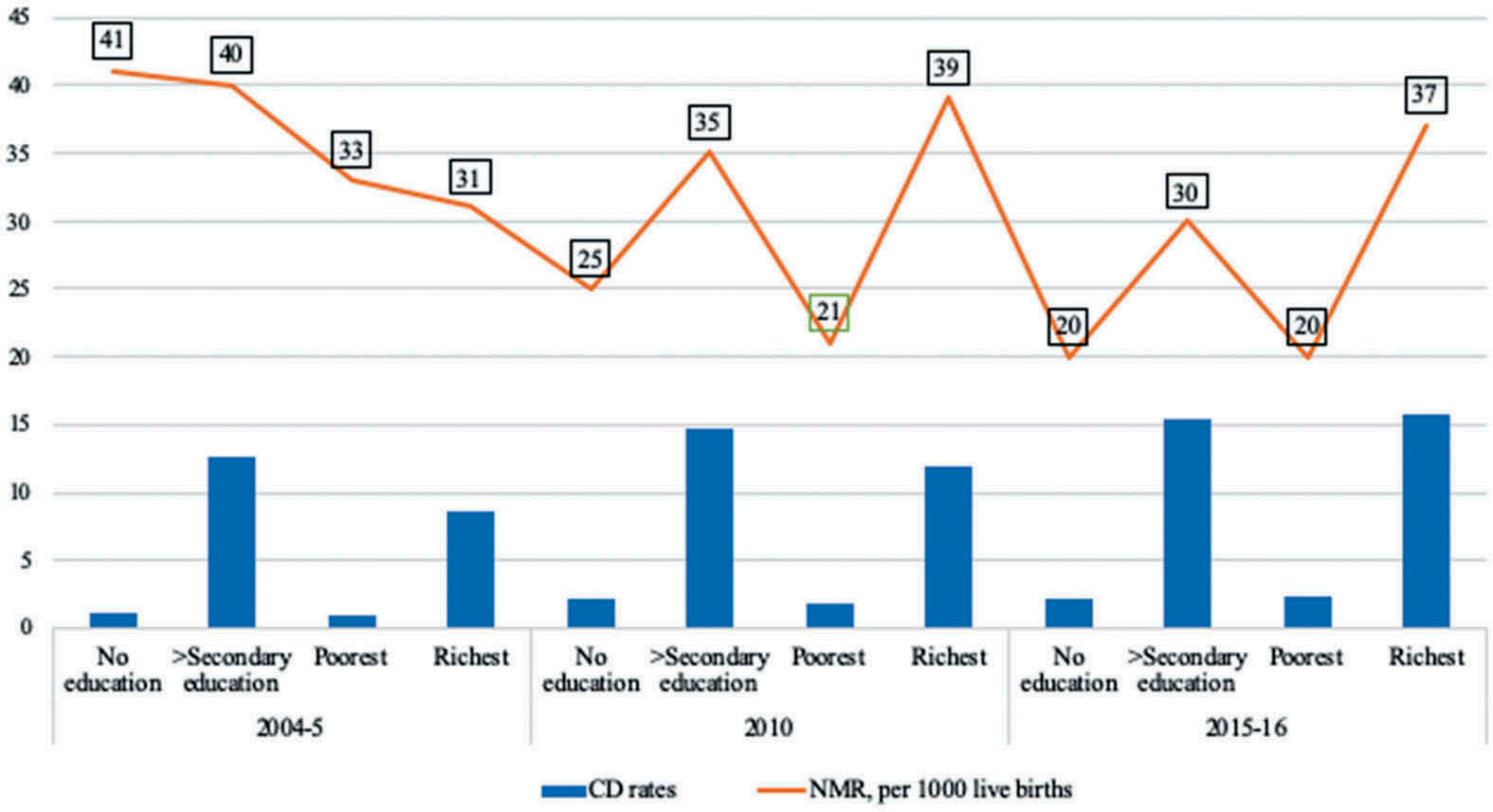
Neonatal mortality rates (NMR) and cesarean delivery (CD) rates among highest and lowest socioeconomic groups in Tanzania between 2004 and 2016 [12,40]

In Kenya and Tanzania where about 100 neonates die daily in each country [10], CD rates among the richest and the secondary+ educated mothers, for Kenya 2014 and Tanzania 2016 indicate an overall difference of more than seven folds higher rates compared to the poorest and the uneducated, respectively, in both countries [11,12]. However, neonatal death rates among the highest SE groups in the two countries were markedly higher compared to those of lowest SE groups. Further since 2003, NMR among the lowest SE categories in Kenya declined by almost half to 20 deaths per 1000 live births in 2014; in contrast, there was almost no overall change in NMRs among the highest SE groups [11,39]. Similar trends can be seen in Tanzania [12,40]. A summary of these reports can be seen in Figures 1 and 2. We identified no population-based studies concerning socioeconomic patterns of CD in relation to NMR in the two countries. A recent global study by Ye et al. investigated the associations between CD rates and NMR accounting for human development index but the study did not adjust for within-country socioeconomic disparities [41]. This study examined the socioeconomic factors associated with cesarean delivery in Kenya and Tanzania. A secondary aim was to assess the impact of cesarean delivery on neonatal survival in both countries.

## Methods

### Study settings

With approximately equal population sizes totaling about 100 million in 2015–2018, Kenya and Tanzania are the most populous countries in the East Africa Community (EAC). Fertility rates were 3.9 − 5 in 2014–2015 [42]. The sex ratio in both countries is 1:1, with women of reproductive age (15–49) comprising roughly 11–12 million in each country [43,44]. More than two-thirds of the populations live in rural areas as farmers [11,12,45]. Maternal health care is free in first-level health centers in both countries, and as a result, institutional births increased to over 60% in recent years [11,12]. In 2015 over 1.5 and 2 million babies were born in Kenya and Tanzania, respectively [10]. In Tanzania, CD rates ranged from 1.1% in the Simiyu region to 17% in Dar es Salaam [12]. In Kenya, CD rates ranged from 2.9% in the northeast to 20.7% in Nairobi [11]. Inadequate financing and equipping of the health facilities are major challenges. A recent assessment in SSA indicated that 18% of health facilities providing CD services did not report presence of any surgical care provider [21].

### Data source and study design

Demographic and Health Survey (DHS) data from Kenya (2014) and Tanzania (2015–2016) with ≥90% response rates were used. We utilized only institutional birth records of the most recent live-born neonates. DHS collects countrywide data on vital reproductive and sociodemographic information in a cross-sectional design. We obtained access to the datasets from DHS secretariat following a written request. The DHS program obtained permission from the host countries, Kenya and Tanzania, to distribute datasets for purposes of health research for common good. The respondents remain completely anonymous and cannot be traced using the data provided. More details on DHS methods of data sampling and collection are publicly available from https://dhsprogram.com/What-We-Do/Survey-Types/DHS-Methodology.cfm.

## Variables

### Outcome and predictor variables

Cesarean delivery (CD) was the main outcome variable for the various socioeconomic variables in the study. Neonatal mortality (NM) was a secondary outcome variable for predictor variable CD. NM was dichotomized as ‘lived’ and ‘died’.

### Maternal and pregnancy-related variables

These constituted potential confounders or explanatory variables that have been hypothesized in many previous studies to be independently associated with either or both CD and neonatal mortality (NM). Major direct causes of NM include sepsis, preterm births, birth asphyxia, and pneumonia [46]. Whereas some of these variables are not direct causes of NM or CD, they are important proxy risk factors and indirect or intermediate risk factors in the causal pathway for both NM and CD. For example, low birthweight is a known underlying risk factor for both preterm and birth asphyxia. Others include; *Maternal age*, which was classified as ‘15–24’, ‘25–34’ and ‘35–49’ years, with age-group 25–34 used as a reference group, the younger and older age-groups have been associated with adverse pregnancy outcomes [47,48]. *Marital status* was dichotomized as ‘single’ and ‘married’ [49]. *Maternal BMI* was categorized as under- and overweight, normal, and obese (non-pregnant and non-postpartum). *Parity* was classified as ‘primiparous’ (first-time mothers), ‘para 2–3’ and ‘para 4+’ [50]. *Newborn sex* and *multiple births* were included, as male sex and multiple gestations have been associated with higher death rates [51,52]. *Number of antenatal care (ANC) visits* was included; higher ANC visits is associated with skilled care and lower neonatal deaths [52]. *ANC* was categorized in terms of detailed as well as broader groupings to examine both current WHO recommendations (>8 visits) [13] and recommendations at the time of data collection (>4 visits), as shown in Tables 1 and 2. *Birthweight* was also included as higher birthweight (>4 kg) and low birthweight (<2.5 kg) are risk factors for both CD and NM, respectively [52,53]. *Facilities of delivery* were included, as private compared to government facilities are associated with CD [54].

**Table 1.** Characteristics of sociodemographic, maternal and newborn variables by cesarean delivery in health-facility births in Kenya 2014 and Tanzania 2015–2016

	Overall, N = 13,372			Kenya (N = 8738)			Tanzania (N = 4634)		
	Cesarean	Normal	P value	Cesarean	Normal	P value	Cesarean	Normal	P value
Characteristics	%	%	95% CI	%	%	95% CI	%	%	95% CI
**Place of residence**									
Rural	9.9	90.1		11.3	88.7		7.8	92.2	
Urban	14.9	85.1	**<0.001**	15.5	84.5	**<0.001**	13.4	86.6	**<0.001**
**Maternal age**									
15–24	9.3	90.7		10.3	89.7		7.3	92.7	
25–34	12.8	87.2		13.6	86.4		11.1	88.9	
35–49	14.4	85.6	**<0.001**	17	83	**<0.001**	10.6	89.4	**<0.01**
**Marital status**				8767					
Single	11.9	88.1		13.6	86.4		8.9	91.2	
Married	12.1	87.9	>0.05	13.2	86.8	>0.05	10	90	>0.05
**Wealth index**									
Poorest	7.8	92.2		8.4	91.6		6.7	93.3	
Poor	9.3	90.7		11.1	88.9		5.2	94.8	
Middle	10.4	89.6		11.7	88.3		7.8	92.2	
Richer	12.1	87.9		13.9	86.1		9.1	90.9	
Richest	17.8	82.2	**<0.001**	18.7	81.3	**<0.001**	16.1	83.9	**<0.001**
**Education level**									
No education	6.9	**93.1**		7.8	92.2		5.9	94.1	
Primary	10.4	**89.6**		11.6	88.4		8.5	91.6	
≥Secondary	15.9	**84.1**	**<0.001**	16.2	83.8	**<0.001**	14.7	85.3	**<0.001**
**Parity**									
Primiparous	14.7	**85.3**		15.7	84.3		12.7	87.3	
Para 2-3	12.9	**87.0**		13.8	86.2		10.9	89.1	
*Mode of delivery data missing, excluded n = 10, P value – chi-square test*									
Para 4+	8.5	**91.5**	**<0.001**	10	90	**<0.001**	6.3	93.7	**<0.001**
**Sex of newborn**									
Male	12.2	87.2	>0.05	13.5	86.5	>0.05	9.5	90.5	>0.05
Female	11.9	88.1		12.9	87.1		10	90	
**Birthweight**									
<2500 g	13.1	86.9		15.1	84.9		11.2	88.8	
2500-4000 g	10.7	89.3		12	88		9.6	90.4	
>4000 g	14	86.0	**<0.01**	16	84	**0.01**	12.1	87.9	**<0.05**
**Multiple births**									
No	11.7	88.3		13	87		9.4	90.6	
Yes	27.1	72.7	**<0.001**	28.1	71.9	**<0.001**	25	75	**<0.001**
**Health facility of birth**									
Gov`t facility	10.3	89.7		11.6	88.4		7.9	92.1	
Mission hospital	19.5	80.5	**<0.001**	19.7	80.3	**<0.001**	18.9	81.1	**<0.001**
Private	–	–	**–**	N/A	N/A		15.7	84.3	**<0.001**
**Antenatal visits**									
0 ANC visits	11.6	88.4	**<0.001**	9.1	90.9	**<0.001**	15.8	84.2	**<0.001**
1–3 visits	9.5	90.5		10.4	89.6		8.2	91.8	
4-7 visits	13.2	86.8		14.5	85.5		10.5	89.5	
8or> visits	23.6	76.4		22.4	77.6		30.9	69.1	
**Antenatal visits II**									
<4 visits	9.6	90.4	**<0.001**	10.3	89.7	**<0.001**	8.4	91.7	**<0.01**
4≥ visits	13.7	86.3		14.9	85.1		10.9	89.1	
**Health insurance**									
No	9.9	90.1		11.1	88.9		8.9	91.1	
*Mode of delivery data missing, excluded n = 10, P value – chi-square test. N/A -not available*									
Yes	18.2	81.8	**<0.001**	18.5	81.5	**<0.001**	17.8	82.3	**<0.001**
*Missing*	*628*	*3907*		*628*	*3907*				
**Occupation**									
Not working	10.3	89.7		11	89		9.3	90.7	
Technical, managerial	21.8	78.2		17.7	82.3		30.9	69.1	
Self-employed farmer	8.6	91.4		11.7	88.3		7.1	92.9	
Domestic service	11.5	88.5	**<0.001**	12.8	87.2	**<0.001**	10.3	89.7	**<0.001**
and manual work									
**Maternal BMI,**									
Underweight, <18.5	8.0	92.0		9.0	91.0		6.5	93.5	
Normal,18.5–24.99	9.1	90.9		10.6	89.4		7.4	92.6	
Overweight, 25–29.99	13.8	86.2		15.1	84.9		11.4	88.6	
Obese, ≥ 30	20.0	80.0	**<0.001**	19.5	80.5	**<0.001**	20.8	79.2	**<0.001**
*Missing*	*695*	*4588*		*667*	*4187*		*28*	*401*	

Mode of delivery data missing, excluded n = 10, P values - from chi-square test at 95% Confidence Interval (CI). All bold values are statistical significant values. All italic values signify missing values.

**Table 2.** Distribution of study variables by neonatal survival outcome in Kenya 2014 and Tanzania 2015–2016

	Overall, N = 12,898			Kenya (N = 8446)			Tanzania (N = 4452)		
Variables/Classification	Died	Lived	P-value 95% CI	Died (%)	Lived (%)	P-value 95% CI	Died (%	Lived (%)	P-value 95% CI
**Cesarean delivery**									
Yes	42(19.2)	1521(12.0)		27 (20.6)	1099(13.2)		15(17.1)	422(9.7)	
No	177(80.8)	11,158(88.0)	**0.001**	104(79.4)	7216(86.8)	**0.01**	73(83)	3942(90.3)	**0.02**
**Place of residence**									
Rural	131(59.8)	7430(58.6)		77(58.8)	4573(55.0)		54(61.4)	2857(65.5)	
Urban	88(40.2)	5249(41.4)	>0.05	54(41.2)	3742(45.0)	>0.05	34(38.6)	1507(34.5)	>0.05
**Maternal age**									
15-24	67(30.6)	3903(30.8)		37(28.2)	2515(30.3)		30(34.1)	1388(31.8)	
25-34	88(40.2)	6079(47.9)		56(42.8)	4193(50.4)		32(36.4)	1886(43.2)	
35-49	64(29.2)	2697(21.3)	**<0.01**	38 [29]	1607(19.3)	**<0.05**	26(29.6)	1090(25.0	>0.05
**Marital status**									
Single	43(19.6)	2251(17.8)		19(14.5)	1427(17.2)		24(27.3)	824(18.9)	**<0.05**
Married	176(80.4)	10,428(82.2)	>0.05	112(85.5)	6888(82.8)	>0.05	64(72.7)	3540(81.1)	
**Wealth index**									
Poor & poorest	71(32.4)	4133(32.6)		50(38.2)	2842(34.2)		21(23.9)	1289(29.5)	
Middle	53(24.2)	2454(19.4)		34 [26]	1641(19.8)		19(21.6)	813(18.6)	
Rich & richest	95(43.4)	6094(48.0)	>0.05	47(35.8)	3832(46.1)	**<0.05**	48(54.6)	2262(51.8)	0.05
**Educational level**									
No education	27(12.3)	1216(9.6)		17(13.0)	629(7.6)		10(11.4)	587(13.5)	
Primary	134(61.2)	6903(54.4)		76(58.0)	4253(51.2)		58(65.9)	2650(60.7)	
≥Secondary	58(26.5)	4560(36.0)	**0.01**	38(29.0)	3433(41.3)	**<0.01**	20(22.7)	1127(25.8)	>0.05
**Parity**									
Primipara	53(24.2)	3551(28.0)		28(21.4)	2363(28.4)		25(28.4)	1188(27.2)	
^a^Mode of delivery data missing, excluded, n^a^ = 10	Overall missing survival status data excluded, n = 503			Missing/excluded, n = 321			Missing/excluded, n = 182		
a – Among babies having survival status information, 10 lacked mode of delivery data and were excluded from analysis. P-values – chi-square									
Para 2-3	79(36.1)	5026(39.6)		48(36.6)	3487(41.9)		31(35.2)	1539(35.3)	
Para 4+	87(39.7)	4102(32.4)	0.05	55(42.0)	2465(29.7)	**0.01**	32(36.4)	1637(37.5)	>0.05
**Sex of newborn**									
Male	123(56.2)	6602(52.1)		67(51.2)	4360(52.4)		56(63.6)	2242(51.4)	
Female	96(43.8)	6077(47.9)	>0.05	64(48.9)	3955(47.6)	>0.05	32(36.4)	2122(48.6)	**<0.05**
**Birthweight**									
<2500 g	25(22.1)	519(6.5)		9(20.9)	264(6.8)		16(22.9)	255(6.2)	
2500–4000 g	70(62.0)	6568(81.9)		26(60.5)	3165(81.5)		44(62.9)	3403(82.3)	
>4000 g	18(15.9)	931(11.6)	**<0.01**	8(18.6)	452(11.7)	**<0.01**	10(14.3)	479(11.6)	**<0.01**
Missing	106	4661		88	4434		18	227	
**Multiple births**									
No	205(93.6)	12,439(98.1)		121(92.4)	8162(98.2)		84(95.5)	4277(98.0)	
Yes	14(6.4)	240(1.9)	**<0.01**	10(7.6)	153(1.9)	**<0.001**	4(4.5)	86(2.0)	>0.05
**Decision for CS**									
Before labor	N/A	N/A		N/A	N/A		3 [20]	120(28.4)	>0.05
After labor	N/A	N/A		N/A	N/A		12(80.0)	302(71.6)	
Missing							73	3942	
**Facility of birth**									
Government facility	177(82.7)	10,082(79.6)		103(81.8)	6508(78.5)		74 (84.1)	3574(81.9)	
Mission hospital	36(16.8)	2430(19.2)	>0.05	23(18.2)	1786(21.5)	>0.05	13 (14.8)	644(14.8)	>0.05
Private	1 (0.5)	146(1.2)					1(1.1)	146(3.3)	
Missing	5	21		5	21				
**Antenatal visits**									
0 ANC visits	11(5.1)	126(1.0)	**<0.001**	9(6.9)	93(1.1)	**<0.001**	2(2.3)	33(0.8)	>0.05
a – Among newborns having survival information, 10 lacked mode of delivery data and were excluded from analysis. P-values from chi-square test. N/A-not available									
1–3 ANC visits	95(43.6)	4797(38.0)		50(38.5)	2919(35.2)		45(51.1)	1878(43.2)	
4–7 ANC visits	108(49.5)	7328(58.0)		67(51.5)	4949(59.8)		41(46.6)	2379(54.8)	
≥8 or ANC visits	4 (1.8)	373(3.0)		4(3.1)	320(3.9)		0(0)	53(1.2)	
Missing	1	55		1	34		0	21	
**Antenatal visits II**				N = 4431					
<4 visits	106(48.6)	4923(39.0)	**<0.01**	59(45.4)	3012(36.4)	**<0.05**	47(53.4)	1911(44.0)	>0.05
4 or more visits	112(51.4)	7701(61.0)		71(54.6)	5269(63.6)		41(46.6)	2432(56.0)	
Missing	1	55		1	34		0	21	
**Health insurance**									
**No**	128 (88.3)	7116(85.0)	>0.05	49(86)	3151(78.7)	>0.05	79(89.8)	3965(90.9)	>0.05
Yes	17(11.7)	1252(15.0)		8(14.0)	853(21.3)		9(10.2)	399(9.1)	
Missing	74	4311		74	4311				
**Maternal BMI**									
Underweight, <18.5	10(8.1)	533(6.9)	>0.05	5(9.8)	262(7.1)	>0.05	5(6.9)	271(6.8)	>0.05
Normal, 18.5–24.99	66(53.7)	4526(58.9)		29(56.9)	2091(56.4)		37(51.4)	2435(61.2)	
Overweight, 25–29.99	27(22.0)	1762(22.9)		12(23.5)	946(25.5)		15(20.8)	816(20.5)	
Obese, ≥ 30	20(16.3)	863(11.2)		5(9.8)	408(11.0)		15(20.8)	455(11.4)	
Missing	96	4995		80	4608		16	387	

^a^Among newborns having survival information, 10 lacked mode of delivery data and were excluded from analysis. P-values from chi-square test.

All bold values indicate statistical significance at 95% confidence Interval (CI).

### Socioeconomic variables

Similarly, the socioeconomic variables included were chosen due to their association with higher CD rates. Thus, urban relative to rural *place of residence* has been associated with higher CD rates [2]. W*ealth, formal occupation*, having *health insurance and* higher *maternal educational levels* have also been associated with higher CD rates of cesarean deliveries in many countries [2,4].

### Data analysis

Analytical software Stata version 12 (College Station, TX: Stata Press.) was used for analysis. Prior to any analysis, we applied sampling weights and adjusted for complex sample design as recommended by the DHS program in order to correct for disproportionate sampling and ensure the population representativeness of the data. Pearson’s chi-square test was used to examine the distribution of study variables. Binomial logistic regression was employed to assess the association between socioeconomic variables and cesarean delivery while controlling for confounders such as maternal age, birthweight, parity and multiple gestations. Similarly, regression analysis was used to examine the association between CD and neonatal mortality with adjustments for confounding at 95% confidence interval.

## Results

Overall, about 13 382 (60%) of mothers delivered in a health facility in Kenya and Tanzania, with similar proportion of institutional births in each country. Table 1 presents the distribution of study variables by mode of delivery. About 13% and 10% of births were through C-section in Kenya and Tanzania, respectively, with overall wider SE disparities in CD rates within the countries. In both countries, socioeconomic status of wealth, higher education level, health insurance and higher maternal occupation were associated with cesarean delivery, p < 0.05. Other factors such as urban residency and use private or mission health facility of birth were also associated with CD in both countries, p < 0.05.

**Figure 3.**
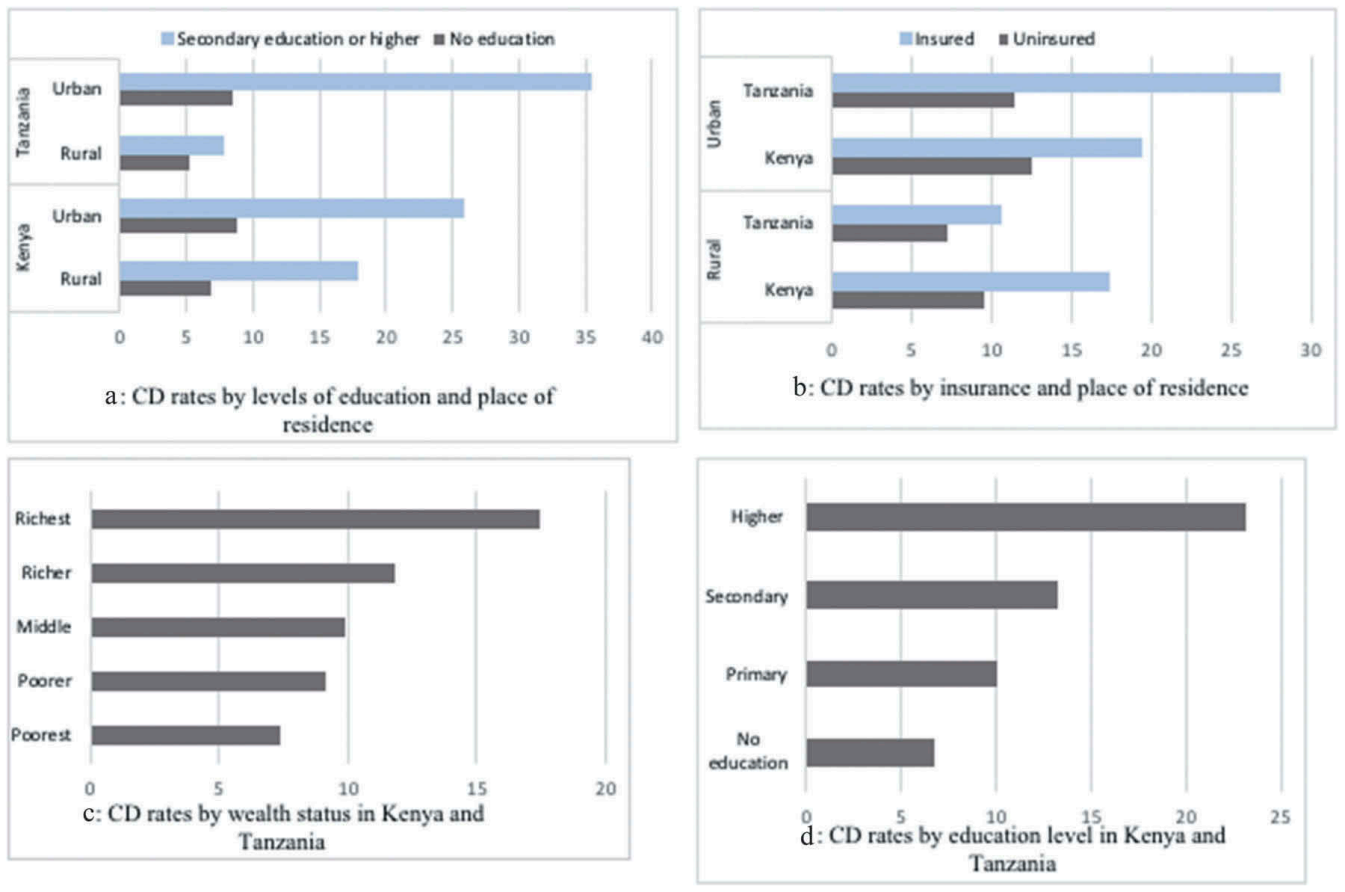
Graphical representations A, B, C, and D showing cesarean delivery rates by socioeconomic characteristics and place of residence in 2014-2016, in Kenya and Tanzania

Table 2 shows the distribution of study variables by neonatal survival in Kenya (2014) and Tanzania (2015–2016). Chi-square test results indicated an association between C-section and neonatal mortality in both countries, p < 0.05. Aggregate analysis also indicated an array of variables that were associated with neonatal mortality including lack of formal education among others, Table 2. A graphical summary of cesarean delivery rates by socioeconomic characteristics and place of delivery is shown in Figure 3 and Table 3. Cesarean delivery rates ranged from 5% among formally uneducated rural women in Tanzania to 26% among highly educated urbanites in Kenya and to 37.5% among urban women in managerial positions in Tanzania. A difference of 19% and 32% between the lowest and highest CD rates in the socioeconomic groups was also observed in Kenya and Tanzania, respectively. Similar, wider disparities in CD trends were shown in both countries on the basis of having health insurance coverage (Figure 3, graph B).

**Table 3.** Within country cesarean section rates, by socioeconomic status, place of delivery and place of residence in Kenya 2014 and Tanzania 2015–16

	Overall, N = 13,372	Kenya, N = 8738		Tanzania = 4634	
	(95% CI)	Rural	Urban	Rural	Urban
**All**	12.0 (11.5–12.6)	11.3	15.5	7.8	13.4
**Wealth status**					
Poorest	7.8 (6.6–9.0)	7.8	10.5	7.2	Missing
Poorer	9.3 (8.1–10.4)	11.2	10.6	5.0	8.6
Middle	10.4 (8.8–9.1)	11.6	11.9	7.9	6.7
Richer	12.1 (11.0–13.2)	13.5	14.2	9.5	8.5
Richest	17.7 (16.4–19.0)	15.6	19.1	1.0	17.5
**Education level**					
No education	6.9 (5.4–8.2)	6.8	8.8	5.3	8.5
Primary	10.4 (9.7–11.1)	10.8	12.9	7.6	10.2
Secondary	13.6 (12.5–14.7)	11.7	15.1	10.3	17.9
Higher	23.5 (21.0–26.0)	17.8	25.8	7.7	35.4
**Maternal occupation**					
Not working	10.3 (9.0–11.6)	10.6	11.3	8.0	10.7
Managerial, technical, clerical	21.8 (18.7–24.8)	14.6	19.7	20.2	37.5
Self-employed farmer	8.6 (7.6–9.6)	11.4	12.7	6.9	8.5
Manual, domestic services	11.5 (10.4–12.6)	10.3	14.9	8.2	12.2
**Health insurance**					
No	9.9 (9.2–10.6)	9.9	12.7	7.5	11.7
Yes	18.2 (16.1–20.3)	17.5	19.2	10.7	29.2
**Health facility of birth**					
Government	10 (9.7–10.8)	10.0	13.7	5.7	11.7
NGO or religious	19.5 (17.9–21.0)	18.4	20.6	18.5	19.9
Private		N/A	N/A	4.2	25.9

**Figure 4.**
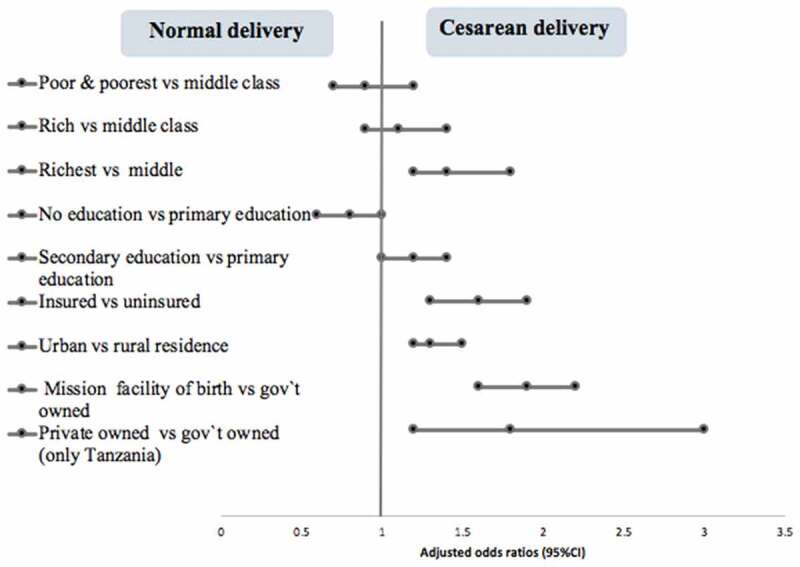
Forest plot presentation of adjusted odds ratios, 95% confidence interval (Table 4), showing aggregate associations between socioeconomic characteristics and cesarean delivery in Kenya and Tanzania, 2014–2016

Table 4 and Figure 4 present adjusted odds ratios for the association between socioeconomic factors and cesarean delivery. Overall findings indicated higher odds of cesarean delivery among mothers from richest households, aOR 1.4 (95% CI 1.2–1.8), those with health insurance, aOR 1.6 (95% CI 1.3–1.9), highly educated, aOR 1.6 (95% CI 1.2–2.0), urban residents, aOR 1.3 (95% CI 1.2–1.5), those in managerial positions, aOR 1.7 (95% CI 1.3–2.2) and among births in mission health facilities, aOR 1.9 (95% CI 1.6–2.2), compared to middle class, no insurance, rural residents, unemployed and government facilities, respectively. Similar trends were observed in Tanzania. However, in Kenya, the higher odds of CD among those with managerial positions and the richest was not statistically significant. Comparatively, the managers and those who delivered in mission hospitals had about 3 times higher odds of cesarean delivery in Tanzania.

**Table 4.** Logistic regression analysis showing associations between socioeconomic factors, place of residence and cesarean delivery in Kenya 2014 and Tanzania, 2015–2016

Overall N = 13,372	Overall	Kenya	Tanzania
Variables	aOR (95% CI)	aOR (95% CI)	aOR (95% CI)
**Wealth status**			
Poorest	0.9(0.7–1.2)	0.8(0.6–1.2)	0.9(0.6–1.4)
Poor	0.9(0.7–1.2)	0.9(0.7–1.2)	0.6(0.4–1.0)
Middle	Ref	Ref	Ref
Rich	1.1(0.9–1.4)	1.1(0.8–1.4)	1.1(0.7–1.4)
Richest	**1.4(1.2–1.8)**	1.2(0.9–1.6)	**1.6(1.2–2.2)**
**Educational level**			
No education	0.8(0.6–1.0)	0.9(0.6–1.4)	0.8(0.5–1.1)
Primary	Ref	Ref	Ref
Secondary	**1.2(1.0–1.4)**	1.1(0.8–1.2)	**1.4(1.1–1.8)**
Higher	**1.6(1.2–2.0)**	**1.4(1.0 − 1.8)**	**2.4(1.3–4.4)**
**Maternal occupation**			
Not working	Ref	Ref	Ref
Managerial, technical, clerical	**1.7(1.3–2.2)**	1.3(0.9–1.7)	**2.9(1.9–4.3)**
Self-employed farmer	0.9(0.7–1.1)	1.0(0.8–1.3)	0.9(0.7–1.3)
Manual, domestic services	1.02(0.84–1.22)	1.0(0.8–1.3)	1.1(0.8–1.5)
**Health Insurance**			
No	Ref	Ref	Ref
Yes	**1.6(1.3–1.9)**	**1.4(1.2–1.8)**	**1.8(1.4–2.4)**
**Place of residence**			
Rural	Ref	Ref	Ref
Urban	**1.3(1.2–1.5)**	**1.2(1.0–1.4)**	**1.5(1.2–1.8)**
**Health facility of birth**			
Government facility	Ref	Ref	Ref
Mission health facility	**1.9(1.6–2.2)**	**1.5(1.2–1.8)**	**2.7(2.1–3.4)**
Private facility	N/A	N/A	**2.2(1.3–3.5)**

Each socioeconomic factor independently adjusted for maternal age, birthweight, parity, multiple births. aOR, adjusted odds ratio. Missing data were excluded from analysis. Bold values indicate statistically significant adjusted odds ratios.

Table 5 shows adjusted odds ratios for the association between cesarean delivery and neonatal mortality. Overall, after controlling for maternal risk factors in Model 1, cesarean delivery had 1.7 times higher odds of neonatal deaths compared to normal births, aggregate aOR 1.7 (95% CI 1.2–2.7). After further adjustments for fetal risk factors in Model 2 and antenatal care visits in model 3, the adjusted OR ceased to be statistically significant, 1.6 (95% CI 0.9–2.6). Aggregate wealth quintile-specific analysis shown in Table 6, adjusted for all Model 3 factors except education level (due to high its correlation with wealth), showed 4.4 folds of higher neonatal mortality among the poorest after cesarean delivery. All other wealth quintiles showed no statistical significance.

**Table 5.** Binomial logistic regression analysis (models 1–3) for the associations between cesarean delivery and neonatal mortality, adjusted odds ratios (aOR) in Kenya 2014 and Tanzania, 2015–2016

	Model 1			Model 2				Model 3		
	aOR (95% CI)			aOR (95% CI)				aOR (95% CI)		
	Overall, N = 12,898	Kenya	Tanzania	Overall	Kenya	Tanzania	Overall		Kenya	Tanzania
**Cesarean section**										
No	Ref	Ref	Ref	Ref	Ref	Ref	Ref		Ref	Ref
Yes	**1.7(1.2–2.7)**	1.6(0.8–3.4)	**1.8(1.0–3.2)**	**1.6(1.0–2.7)**	1.5(0.7–3.5)	1.7(0.9–3.4)	1.6(0.9–2.6)		1.4(0.6–3.2)	1.7(0.9–3.4)

Model 1: Adjusted for maternal factors (Maternal age, parity, education level and BMI) Model 2: Model 1 factors and fetal risk factors (multiple births and birthweight), Model 3: Models 1 & 2 factors and number of antenatal visits. Bold values indicate statistically significant odds ratios.

**Table 6.** Wealth quintile-specific logistic regression for the association between cesarean delivery and neonatal mortality in Kenya and Tanzania, 2014–2016

Wealth quintiles	Adjusted odds ratios (95% CI)
Poorest (n = 1044)	**4.4 (1.2–16.3)**
Poor (n = 1313)	1.0 (0.1–7.8)
Middle (n = 1528)	0.5 (0.1–2.3)
Rich (n = 2025)	2.4 (0.9–6.3)
Richest (n = 2014)	1.3 (0.5–3.4)

Adjusted for maternal factors (maternal age, parity, BMI, excluding education), fetal risk factors (multiple births and birthweight) and number of antenatal visits. Missing data were excluded from analysis

## Discussion

Overall, our study found that cesarean delivery in Kenya and Tanzania was associated with higher socioeconomic status, indicating that the rising cesarean births might not necessarily be driven by only medical indication, as advised by the WHO. After adjusting for potential confounders, the richest, the highly educated, the insured, managers, urban residents and those who delivered in mission or private facilities comparatively had about 1.4–1.9 times higher odds of cesarean delivery. These findings are in agreement with other studies from LMIC [2,4]. Compared to normal births, cesarean delivery also indicated association with neonatal mortality; however, after further adjusting for key confounders, the findings ceased to be statistically significant. Nonetheless, wealth quintile-specific analysis further indicated that the poorest had the highest odds (OR,4.4) of cesarean-related neonatal deaths even though they had the lowest cesarean delivery rates. These findings partly concur with previous health facility-based studies across many low-and middle-income settings that suggest that cesarean delivery (CD), both emergency and planned, has had net poor perinatal and neonatal outcomes [5,22–28]

This study is perhaps the first of its kind to examine the influence of socioeconomic factors on cesarean delivery and neonatal survival outcome resulting from C-section at national levels in Kenya and Tanzania. C-section as an increasingly preferred mode of birth does not guarantee better neonatal outcomes in East Africa. These findings suggest that a comprehensive evaluation of the rising CD-decisions is needed. Medical indication [9] and maternal informed choice after counseling should be the only basis for cesarean delivery. Other influencing factors such as financial gains should not be an underlying factor for a CD-decision. Streamlining of policies for safe delivery such as comprehensive implementation of practical guidelines including Robson 10-group classifications and recording of delivery decisions and outcomes ought to be implemented at all levels of health institutions in Kenya and Tanzania. The policies should also address delays to seek or receive care and fears of litigations [33,34,55]. Factors surrounding CD appear to be multifaceted and complex in low-resourced health systems in Kenya and Tanzania. However, with existing evidence-based research on CD and recommendations based on increasing research evidence at population levels, rapid progress in policy development and subsequent reduction in CD-related inequities and mortalities can be realized.

A good indication of progress was that even after controlling for only maternal factors, the odds of neonatal mortality following CD in Kenya was not statistically significant. Although our study does not ascertain whether or not neonatal deaths occurred as a result of cesarean procedure itself or due to fetal or pregnancy complications or both, it nonetheless reveals that irrespective of whether there was a medical indication or not, CD-born neonates had higher odds of mortality, among the poorest and overall in Tanzania when only maternal risk factors were adjusted for. Supportive of these findings, another most recent cohort study in *The Lancet* found that neonatal deaths after CD in Africa were double the global NM estimate and maternal deaths after CD were 50 times higher in LMIC in Africa relative to HIC. The study cited anesthesia complications and peripartum hemorrhage as major risk factors [56].

Disparities in adverse neonatal outcomes due to socioeconomic inequities in Kenya and Tanzania appear to diminish over the years and that can be attributed to improved access to health care among the poor and partly due to the slowly declining neonatal death rates among the wealthy as compared to the poor. Recent rising access to C-section associated with higher socioeconomic groups in east Africa [10–12] does not seem to achieve corresponding improved neonatal outcomes. Review of resource allocations and cost-effectiveness in maternity care in these low-resourced health systems could save resources for better neonatal and pregnancy outcomes. Whereas the choice and safety of CD could be well addressed at individual and health facility levels, multifaceted and holistic approaches could improve equitable access and neonatal outcomes. CD on medical grounds and/or well-informed choice (counseling) with zero economic advantage can be positively impactful. Additionally, at administrative levels, mandatory recording of mode of delivery and neonatal outcomes at facility levels could enable continuous auditing, monitoring, and accountability. At community levels, sexual and reproductive health education could ease the burden in the health systems through eliminating unplanned pregnancies, curb delays to seek care, and minimize CD risks. At district and county levels, continuous and equitable allocation of funds to health facilities together with requirements for accountability would improve access. Nationally, continuous training of new health personnel including anesthesiologists and capacity development of existing cadres using the most-updated evidence-based practices would ensure improved quality of cesarean procedures.

Contrary to our findings, elsewhere in Nepal, a country with similar economic conditions as Tanzania but considerably lower gross domestic product per capita compared to Kenya, a significant reduction of neonatal mortalities, including CD-related, has been found [57]. To highlight the difference, for instance, a comparison can be made between two parallel studies [58,59] from matching district-level hospitals with similar year of data collection and numbers of cesarean deliveries (330 vs 327) in Kenya [58] and Nepal [59]. The majority (43%) of patients in Nepal hospitals were of disadvantaged lower caste comparable to patients in the refugees` area in northeastern Kenya. The studies reported 7.3% vs 1.5% neonatal deaths in Kenya and Nepal, respectively.

Considering the 10 years countdown to SDG 2030, to accelerate improved equitable access and better CD-related neonatal survival, we suggest three more approaches. In addition to Betran et al.’s 2018 [55] recommendations of educational interventions for expectant mothers, effective leadership, training of health workforce, adequate equipping and financing, removal of economic incentives for CD and quenching fears of litigations, we suggest the following. Firstly that the National Road Map Strategic Plans for Maternal, Newborn Health and the decentralized health commissions in Tanzania and Kenya should consider adopting the much stronger community-level frameworks that have shown nationwide success through accountability and pregnancy-related support for women in Nepal [57] and Rwanda [60]. Even if all pregnant women accessed hospitals in Kenya and Tanzania, the health-care system would be insufficient to care for them all, much less the C-section cases. Thus, secondly, we suggest strengthening sexual and reproductive education to prevent unplanned pregnancies especially among teenage girls. Thirdly, we proposed mandatory recording of birth, newborns' health and mortality information at the health facilities to enable effective and continuous research, monitoring and accountability.

## Methodological considerations

Over 60% of births in Kenya and Tanzania were institutional, an increase of over 10% from previous years. In addition, the response rate for women interviewed in the DHS program was over 90% for both countries. This improved the analytical power, external validity and representativeness of our findings. Furthermore, the random sampling strategy of DHS data collection minimized selection bias. We also applied sample weights and adjusted for complex sampling design to improve internal validity and representativeness of our sample. Our study found evidence of associations; however, causal interpretation cannot be inferred due to lack of medical confirmation of the actual reason for CD and the cause(s) of neonatal deaths. Our data could also not differentiate between cesarean deliveries that were planned (or elective) or emergency. A key limitation to our study is the many missing survival outcome status of health-facility born babies, the missing, n (503) could have perhaps altered our results if they were not uniformly distributed across. Recall bias as a limitation in cross-sectional design could not be entirely ruled out in our study; however, reproductive events are of significance to women and evidence of accurate recall has been reported [61]. Further, we used the most recent birth data which minimized recall bias. Also, we used non-pregnancy and non-postpartum BMI rather than the actual BMI before and at delivery time, which may have limited our accuracy.

## Conclusion

Disproportionate access to C-section in Kenya and Tanzania is widening along socioeconomic disparity lines. Higher risks of cesarean-related neonatal mortality exist. Choice and/or safety of cesarean delivery can best be addressed on individual basis at health-facility levels. Policy improvements to promote holistic approaches of equitable access on medical grounds as well as informed choice to reduce unnecessary C-sections is vital. Moving forward, reproductive health education to minimize unintended pregnancies, mandatory recording of birth, health and death information for continuous research, monitoring and accountability could improve overall neonatal outcomes. Equipping of health facilities, training and continuous capacity development of health workers to enhance safe delivery services are vital.

## Paper context

The increasing trends of cesarean delivery in Kenya and Tanzania is well known. However, no national peer-reviewed studies in our knowledge have examined the socioeconomic disparities in access and the impact of increasing trends of cesarean sections on the survival of neonates in these low-resourced countries. In this study, we aimed to examine utilization and neonatal outcomes of cesarean delivery with the hope to inform policy to improve newborn survival in Kenya and Tanzania.
